# Monodisperse palladium–cobalt alloy nanocatalyst supported on activated carbon (AC) as highly effective catalyst for the DMAB dehydrocoupling

**DOI:** 10.1038/s41598-020-68773-x

**Published:** 2020-07-16

**Authors:** Betul Sen, Hilal Acidereli, Neslihan Karaman, Fatih Sen

**Affiliations:** Sen Research Group, Biochemistry Department, Faculty of Arts and Science, Dumlupınar University, Evliya Celebi Campus, 43100 Kutahya, Turkey

**Keywords:** Hydrogen storage materials, Hydrology

## Abstract

In the study, activated carbon (AC) supported palladium/cobalt (Pd/Co) nanocatalyst was synthesized to achieve hydrogen release from dimethylamine boron (DMAB). Nanocatalyst were produced by the reduction of Pd^2+^ and Co^2+^ cations by the ultrasonic double reduction method. Analytical studies of the synthesized nanomaterials were characterized by X-ray photoelectron spectroscopy, Raman spectroscopy, X-ray diffraction, transmission electron microscopy (TEM), high-resolution transmission electron microscopy (HR-TEM), electron energy loss spectroscopy, ultraviolet–visible spectroscopy. In this research, nanomaterials exhibited high catalytic activity and reusability, and great performance at low temperatures and concentrations. For the dehydrogenation reaction of dimethylamine borane, TOF and Ea were calculated as 379.5 h^−1^ and 75.86 kJ mol^−1^, respectively. The PdCo@AC nanocatalyst can be used as a promising catalyst for the hydrogen production reaction from DMAB.

## Introduction

Energy is considered a component of all activities and plays an important role in the economic development of any country. Increasing energy demand of the end users, one of the biggest problems of today, have been led researchers to investigate alternative, renewable and clean energy sources for energy supplying and storage. The use of hydrogen in countries with high energy dependence is widely involved in research and development. The hydrogen energy system is the best energy system expected to replace the fossil fuel system before the end of the twenty-first century. Hydrogen; in the next decades, is expected to become widespread use as an energy carrier for the supply of cheap electricity generation. However, easy production and improvement of storage are key points of the hydrogen economy for being safe, efficient and economical. Hydrogen energy is a high quality, non-polluting and, environmentally friendly. The state of art in the hydrogen economy are large and impressive. The creation of a worldwide energy network is essential for safe, economical transport^1–4^.

To date, metal complexes, metallic alloys, metal–organic frames (MOFs), and carbon materials have been studied as hydrogen storage materials. Metals such as Pd, Ru, Pt, Rh have been investigated for hydrogen storage. Since Pd can absorb a thousand times its own volume of hydrogen at ambient temperature and pressure, it is one of the leading hydrogen storage material. Therefore, research on Pd hydride (Pd-H) has been extensively studied in various fields, including hydrogen storage materials, purification filters, isotope separation membranes, and sensors. Such metals show high performance for hydrogen storage^5, 6^. However, its high-cost is big a disadvantage. In order to reduce the cost, studies have been performed with low-cost metal nanocatalysts such as nickel (Ni), copper (Cu), iron (Fe), and cobalt (Co). When cost effective metals are used alone, catalyst showed low performance and reusability^7–9^. For this reason, it is aimed to synthesize both high activity and relatively cost effective catalyst by forming Co–Pd alloy. Activated carbon (AC) was used as a support to increase the active surface area of metal alloy and improve the catalytic performance. The AC support dimensionally stabilizes Pd/Co metal alloy and has great potential as hydrogen carriers.

The ammonia boranes (AB) and its derivatives are a good source of hydrogen in the presence of a suitable catalyst and have a high hydrogen content. Under optimal conditions and suitable catalyst, hydrogen can be produced from the ABs^10, 11^. Dimethylamine borane (DMAB) is an environmentally friendly, stable AB derivative. The disadvantage of dehydrogenation of AB is the long induction time before the start of hydrogen release^12^. Safe and efficient hydrogen storage is possible with new generation technologies. However, still there is a need to develop industrial-scale low-cost nanocatalysts for the dehydrogenation of the ABs and its derivatives and, transport is a problem due to the low hydrogen density. Therefore, the need for high-density chemicals to transport hydrogen has increased^13, 14^. To this end, hydrazine (N_2_H_4_), ammonia-borane (NH_3_BH_3_), sodium borohydride (NaBH_4_) are used^15–18^. Several different support materials and metals are important components in the composition of catalysts to obtain highly active and stable nanocatalyst. DMAB from AB derivatives is preferred because of its properties such as crystalline solid, stable in air and water, non-toxic. Compared to ABs, DMAB products are a good model substrate that is easy to maintain and control^19^.

Equation (1) shows the catalytic reaction for the release of hydrogen from DMAB. When 1 mol of DMAB reacts, 1 mol of hydrogen gas is released. The reaction is conducted in room conditions (RT).

1 $${2}\left( {{\text{CH}}_{{3}} } \right)_{{2}} {\text{NHBH}}_{{3}} \mathop{\longrightarrow}\limits_{{{\text{RT}}}}^{{{\text{Catalyst}}}}\left[ {\left( {{\text{CH}}_{{3}} } \right)_{{2}} {\text{N}} \cdot {\text{BH}}_{{2}} } \right)]_{{2}} + {\text{ 2H}}_{{{2} }}$$

Dimethylamine borane dehydrogenation reaction [Cp_2_Ti], Rh4-6 clusters, RhCl_3_, colloidal Rh/[Oct_4_N) Cl, iridium, palladium, rhodium, ruthenium complexes, Rh/Al_2_0_3_, Pt (0)/amyl amine, laurate-stabilized Rh (0), Re complexes, Ru/ZIF-8, Pd (0)/MOF catalysts were tested^19–28^. Synthesis and characterization of palladium/cobalt nanomaterials stabilized with activated carbon on the release of hydrogen from DMAB were performed. The Pd/Co metal was reduced together using the ultrasonic double reduction method. The activated carbon used as a support material in the synthesized catalyst is a carbonaceous material with a well-developed porous structure and large surface area. This is to give the activated carbon a strong adsorption property. The structure of the PdCo@AC nanomaterial was characterized by XPS, XRD, Raman, TEM, and HRTEM.

## Materials and methods

### Synthesis of activated carbon stabilized palladium/cobalt nanocatalyst

In this research, PdCo@AC nanocatalyst was successfully synthesized by the ultrasonic double reduction method^29^. 0.25 mmol of CoCl_2_ and 0.25 mmol of PdCl_2_ were stirred under ultrasonic conditions by adding 15 mL tetrahydrofuran (THF) and 2.5 mmol of activated carbon was added. The mixture was refluxed for two hours at 90 °C. The catalyst was synthesized using the Pd/Co nanocatalyst support material activated carbon to form a blackish solid material. The formation of black color is indicative of a stable nanocatalyst. Activated carbon was used as a support material. Stability, reusability catalytic performances of PdCo@AC nanocatalyst synthesized for DMAB dehydrogenation reaction were investigated. The experiments were performed in three parallel, and data presented in graphics is average of triplicated data. Error bars represented standard deviation (SD). Materials and equipment used are given in Supporting Information.

## Results and discussions

### Synthesis and analytical characterization of PdCo@AC nanocatalyst

**Figure 1:**
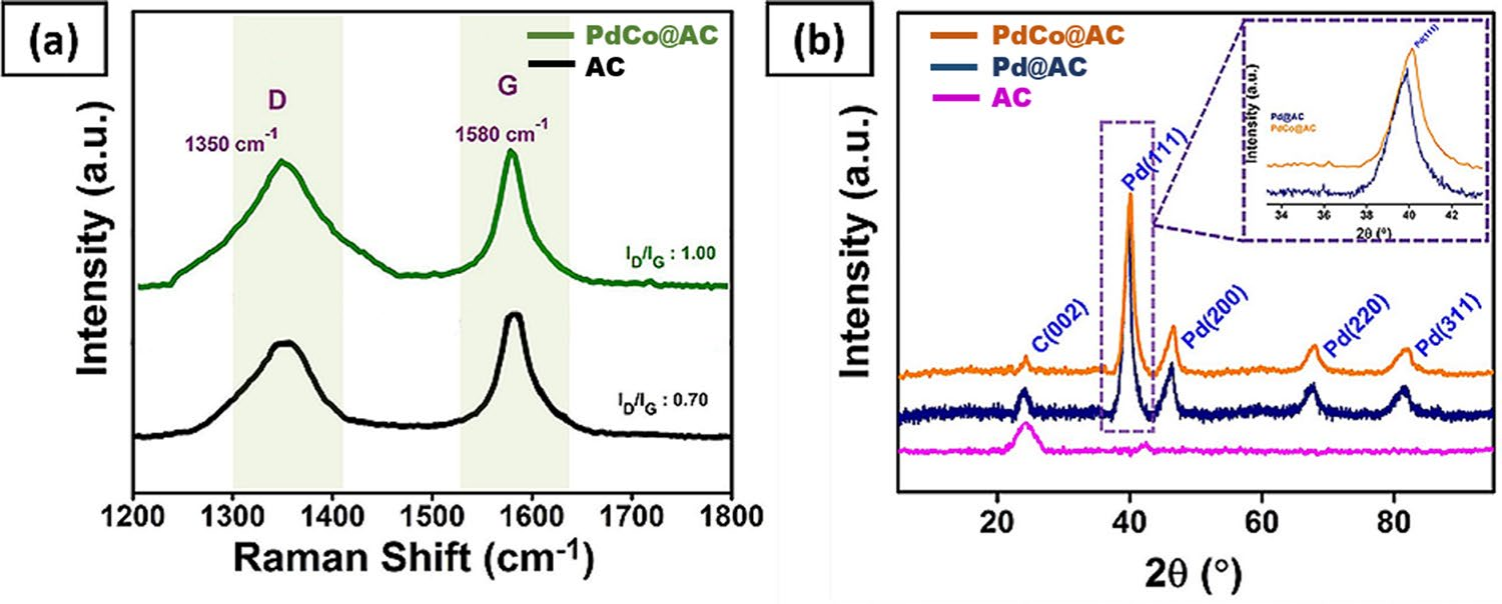
(**a**) Raman spectrum of AC and PdCo@AC; (**b**) XRD of AC, Pd@AC and PdCo@AC NPs.

In this study, for carbonaceous materials were identified by the Raman spectroscopy. The Raman spectrum of PdCo@AC nanocatalyst and activated carbon is shown in Fig. 1a. In Raman spectroscopy, the scattering peaks were detected 1,350.6 cm^−1^ and 1,580.8 cm^−1^. The I_D_/I_G_ ratio can be used for the degree of modification in AC. I_D_/I_G_ ratios of AC and PdCo@AC nanocatalyst were calculated as 0.70 and 1.00, respectively, indicating that the activated carbon (AC) lattice deteriorated after functionalization by palladium and cobalt metals.

XRD technique was performed to identify the crystal structure and average crystallite size of PdCo@AC nanocatalyst. In Fig. 1b, the peaks at 2θ = 40.2°, 48.6°, 71.7°, and 86.6°corresponding to the crystal planes of Pd (111), (200), (220) and (311), respectively, that indicate face-centered cubic structure. Moreover, diffraction peaks shifted slightly to higher 2θ values compared to pure palladium. This indicates the alloy formation of PdCo@AC nanocatalyst. Here, around 24.9° peak is related to activated carbon. In the X-ray powder diffraction models for Pd/Co, the diffraction peaks of the cobalt species were not significant due to the strong signals for the palladium species associated with the amorphous structure of the materials. The crystalline size was calculated as 3.71 ± 0.43 nm using Eq. (2)^30^.

2 $$d\left(\AA \right)=\frac{k\lambda }{\beta cos\theta }$$

where λ is the wavelength of the X-ray used (1.54056 Ǻ), k coefficient (0.9), β is the full width of the half-width of the respective diffraction peak in rad radiation, and θ is the maximum peak in rad position.

The lattice parameter was calculated using Pd (220) diffraction peak. The lattice parameter value for PdCo@AC nanocatalyst was calculated as 3.88 Å using Eq. (3). This result is in line with the pure palladium value (3.89 Å)^21, 31, 32^.

3 $$Sin \theta =\frac{\lambda \sqrt{{h}^{2}+{k}^{2}{+l}^{2}}}{2a}$$

According to the UV–VIS results, as shown in SI Fig. S1, the conversion of the palladium and cobalt material to the Pd/Co nanomaterials with the activated carbon support material is large and the absorption lines indicating that the cations at the reflux end are reduced due to transitions of d–d disappeared.

**Figure 2:**
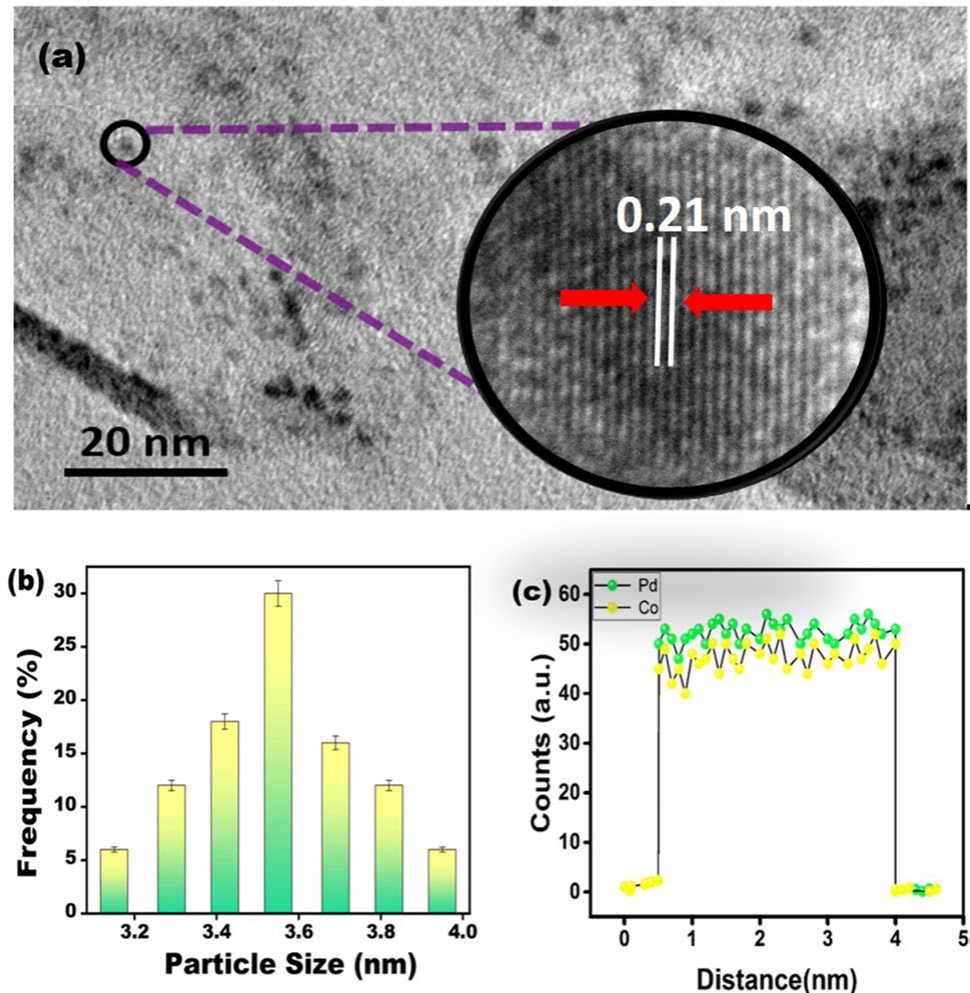
(**a**) TEM and HR-TEM image, (**b**) particle size histogram, (**c**) the EELS line profile of the Pd/Co nanocatalyst.

In Fig. 2, the morphology, composition and particle size of PdCo@AC nanocatalyst were determined by TEM-ELLS analyses. The HR-TEM results showing the morphology of the nanomaterial are shown in Fig. 2a. Particle size of 3.55 ± 0.40 nm was measured as shown in Fig. 2b. The particles were spherical and there was no agglomeration. In the HRTEM image, atomic lattice fringes of PdCo@AC nanocatalyst were also observed. The atomic lattice fringe of Pd (111) plane was determined as 0.21 nm which is smaller than the nominal Pd (111) range (0.22 nm)^12, 32–34^. This result indicates that particle size is reduced by formation of Pd–Co alloy. Figure 2c shows the EELS line profile of the Pd–Co nanoparticles. Accordingly, Pd and Co forming an alloy of about 1:1.

**Figure 3:**
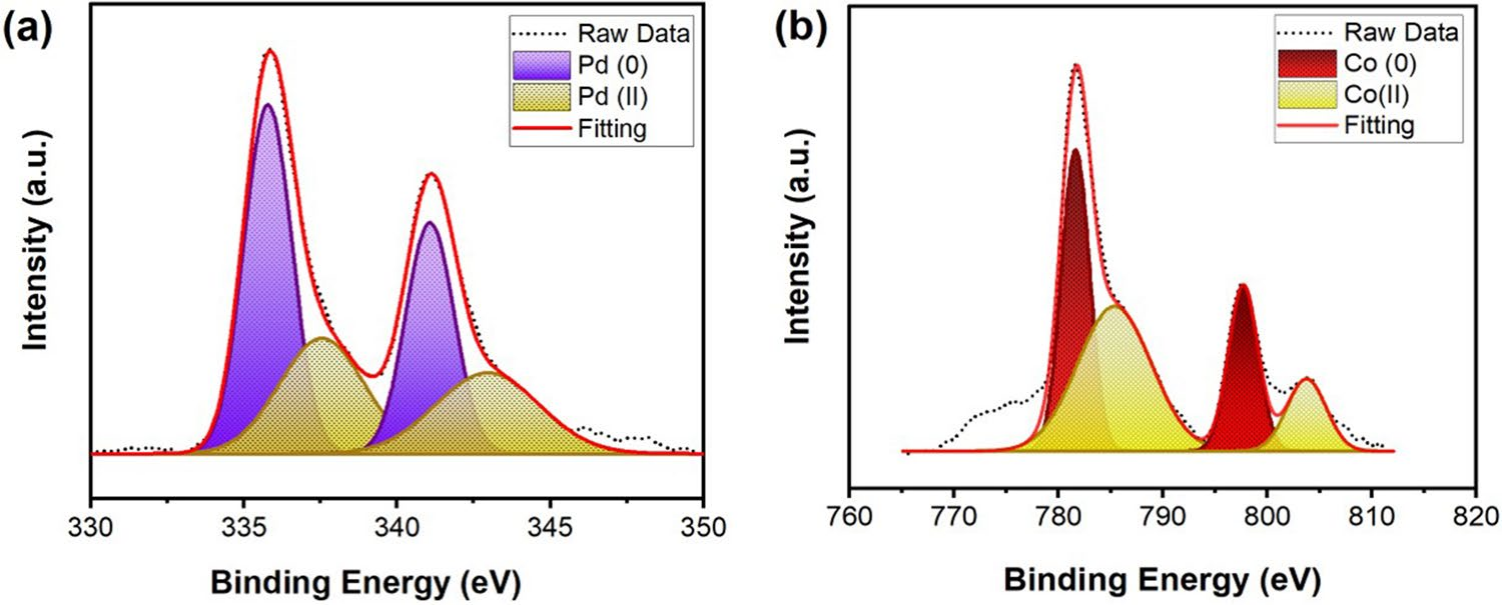
(**a**) Palladium 3d (**b**) Cobalt 2p XPS spectra of PdCo@AC nanocatalyst.

Surface composition and chemical oxidation states of palladium and cobalt in PdCo@AC nanocatalyst were investigated by XPS analytical method. The spectra of Pd (3d) and Co (2p) regions were determined by the Gaussian–Lorentz method. In the X-ray photoelectron spectroscopy spectrum, the binding energies were determined with reference to the 284.1 eV C1s peak. The XPS spectrum, metallic Pd (0) peaks were seen at 335.7 eV and 341.0 eV and Pd (II) peaks were detected at 337.5 eV and 343.0 eV (Fig. 3a). Co (0) peaks were seen at 781.6 eV and 797.6 eV and Co (II) peaks were detected at 785.4 eV and 803.7 eV (Fig. 3b). Comparison of peak intensities revealed that Pd and Co are mostly in metallic form. The low amount of oxidation peaks due to the fact that oxidation of environmental oxygen may result chemical absorption on the surface.

During the catalytic dehydrogenation reaction of DMAB, it was observed that metal composites of palladium and cobalt nanocatalyst were physically mixed (1:1) and Pd/Co bimetallic nanocatalyst (1:1) was synthesized successfully. PdCo@AC nanocatalyst has higher catalytic performance than pure palladium and cobalt mixtures due to the synergetic effect of each component. NMR analysis results for (CH_3_)_2_NHBH_3_ are given in Supporting Information.

### Investigation of the performance of palladium/cobalt nanomaterials stabilized with activated carbon support

**Figure 4:**
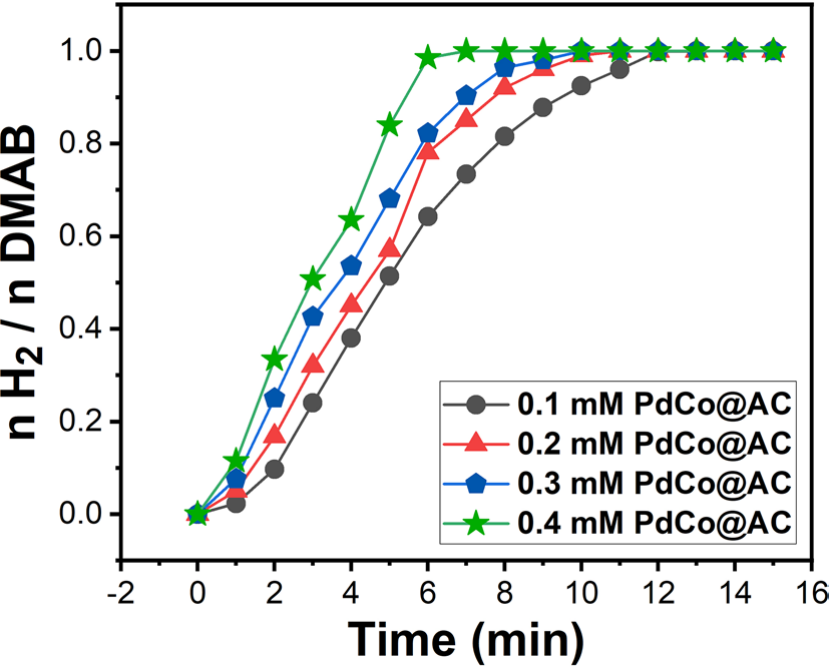
(**a**) Plot nH_2_/nDMAB versus time for dehydrogenation of DMAB in the presence of PdCo@AC nanocatalyst at different concentrations at 25.0 ± 0.1 °C.

In this research, the PdCo@AC nanocatalyst can be used as high-efficiency catalysts on the release of hydrogen from dimethylamine borane. The nH_2_/nDMAB plot versus time is shown at 25.0 ± 0.10 °C at different concentrations of 0.1 mM, 0.2 mM, 0.3 mM and 0.4 mM in Fig. 4a for the dehydrogenation reaction of dimethylamine borane. The release of hydrogen starts timely, induction linearly and rapidly, and continues until the release of hydrogen from dimethylamine borane ends.

**Figure 5:**
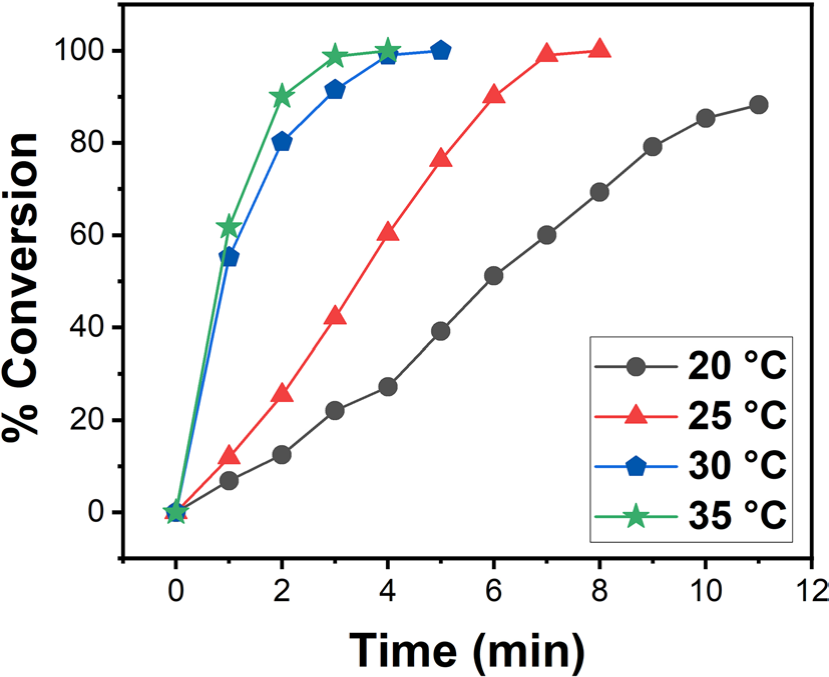
Plot of conversion percentage versus time for DMAB (150 mM in 15 mL THF) beginning with PdCo@AC NPs ([PdCo@AC NPs] 7.5 mol % in THF) at varied temperatures within 20–35 °C range.

**Figure 6:**
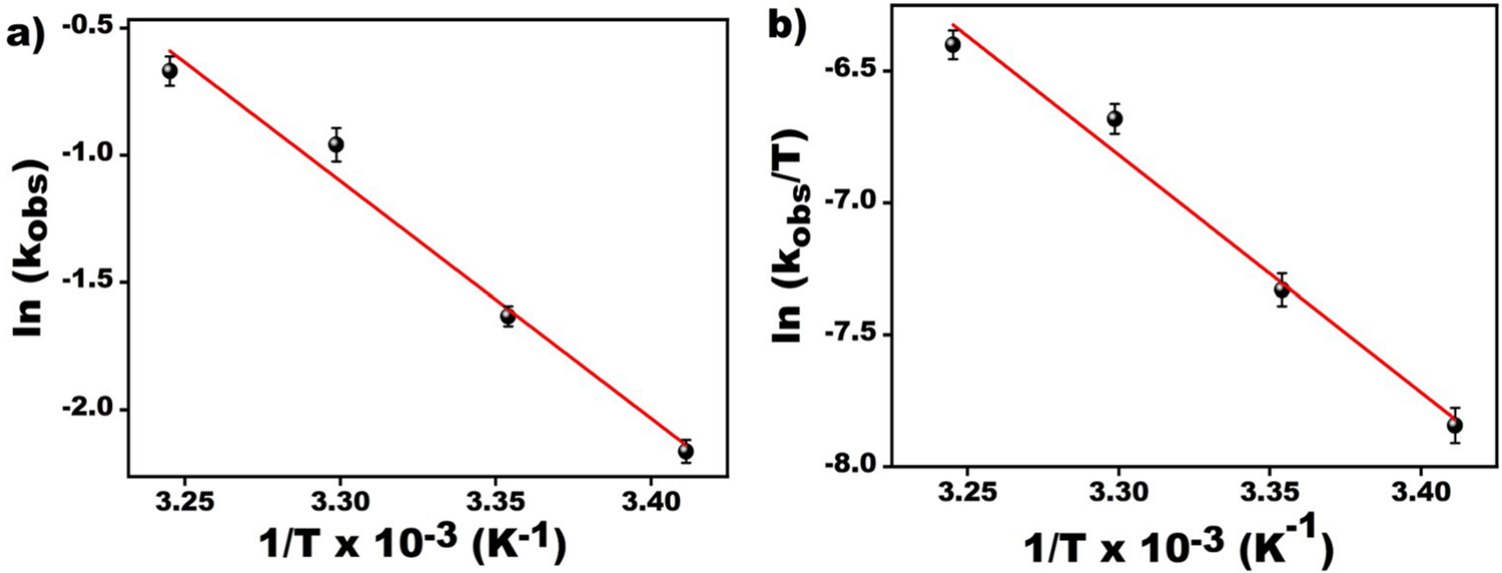
(**a**) Arrhenius and (**b**) eyring plots for the PdCo@AC nanocatalyst dehydrogenation of DMAB at various temperatures.

Hydrogen production rate constants at different temperatures are determined and plotted in Fig. 5. Arrhenius and Eyring plots were drawn with the help of Fig. 5, and the TOF, Ea, ∆H, and ∆S values were calculated. Figure 6a shows the activation energy that calculated as 75.86 kJ mol^−1^ using Arrhenius plot rate constants. With the help of Fig. 6b, ∆H and ∆S were calculated as 73.36 kJ mol^−1^ and − 12.37 J mol^−1^ K^−1^, respectively. The percent conversion calculated after the three parallel experiments were given in the Supporting Information.

TOF value of the PdCo@AC nanocatalyst was calculated as 379.5 h^−1^. TOF value (379.5 h^−1^) is one of the highest values in the literature. Table 1 summarizes that some different nanocatalysts were tested for hydrogen production from DMAB. As shown in Table 1, some bimetallic catalysts exhibited high catalytic performance and TOF values in the same reaction. The result showed that PdCo@AC nanocatalyst can be used effectively in catalytic dehydrogenation reactions due to its efficiency, stability and reusability.

**Table 1:** TOF values of catalysts for dimethylamine borane dehydrogenation.

Catalysts	TOF (h^−1^)	Conversion (%)	References
PdCo@AC	379.5	100	This study
Ru(cod)(cot)	1.6	40	^28^
Cp_2_Ti	12.3	100	^35^
[Rh(1,5-cod)_2_]Otf	12.0	95	^36^
[RhCl(PHCy_2_)_3_]	2.6	100	^22^
[Cr(CO)_5_(thf)]	13.4	97	^21^
[Cp*Rh(m-Cl)Cl]_2_	0.9	100	^37^
PdCo@PVP	330	100	^32^
HRh(CO)(PPh_3_)_3_	0.1	5	^38^
RuCl_3_·3H_2_O	2.7	77	^35^
trans-RuMe_2_(PMe_3_)_4_	12.4	100	^36^
[Rh(1,5-cod)m-Cl]_2_	12.5	100	^36^
[Ru(1,5-cod)Cl_2_]n	2.5	70	^35^
Pt(0)/TBA	31.24	100	^39^
RhCl(PPh_3_)_3_	4.3	100	^36^
IrCl_3_	0.3	25	^38^
Pd/C	2.8	95	^40^
RhCl_3_	7.9	90	^41^
[Cr(CO)_5_(ɳ1-BH_3_NMe_3_)]	19.9	97	^21^
[RuH(PMe_3_)(NC_2_H_4_PPr_2_)_2_]	1.5	100	^38^
Ni(skeletal)	3.2	100	^23^
[Ir(1,5-cod)m-Cl]_2_	0.7	95	^42^
Pt(0)/TPA@AC	34.14	100	^39^
[Rh(1,5-cod)(dmpe)]PF_6_	1.7	95	^40^
(Idipp)CuCl	0.3	100	^43^
Rh(0)NPs	60.0	100	^44^

The cyclic property of activated carbon-supported Pd/Co nanocatalyst was investigated. As shown in SI Fig. S2, the PdCo@AC nanocatalyst retained about 80% of initial performance after the 7th cycle. The diminution of catalytic activity in the dimethylamine borane dehydrogenation reaction is due to the increased amount of nanocatalyst on the surfaces of the nanocatalyst, which may result in passivation and therefore reduced active site accessibility.

## Conclusions

- The active carbon-supported Pd/Co nanomaterials upon hydrogen release from dimethylamine borane were synthesized as an effective nanocatalyst PdCo@AC by ultrasonic double reduction.
- Synthesized PdCo@AC nanocatalyst exhibited very good catalytic performance compared to literature data for dehydrogenation of DMAB.
- The TOF value was found to be 379.5 h^−1^ on the hydrogen release reaction from DMAB.
- Activation energy, ∆H, and ∆S in the presence of PdCo@AC nanocatalyst were calculated as 75.86 kJ mol^−1^, 73.36 kJ mol^−1^, and − 12.37 J mol^−1^ K^−1^, respectively.
- Results showed that PdCo@AC nanocatalyst is promising nanomaterials for the dehydrogenation of DMAB and can be utilized for the hydrogen energy applications.

## Supplementary information

Supplementary file1 (DOCX 289 kb)
