# Supplementary material for: Monodisperse palladium–cobalt alloy nanocatalyst supported on activated carbon (AC) as highly effective catalyst for the DMAB dehydrocoupling
Source: Sci Rep. 2020 Jul 16;10:11755. doi: 10.1038/s41598-020-68773-x (PMC7366684; doi:10.1038/s41598-020-68773-x)
Supplement: Supplementary file 1 — Supplementary file1 (DOCX 289 kb) [file 41598_2020_68773_MOESM1_ESM.docx]

**Supplementary Information**

**Monodisperse Palladium-Cobalt Alloy Nanocatalyst Supported on Activated Carbon (AC) as Highly Effective Catalyst for the DMAB dehydrocoupling**

Betul Sen^1^, Hilal Acidereli^1^, Neslihan Karaman, Fatih Sen*

*^a*^Sen Research Group, Biochemistry Department, Faculty of Arts and Science, Dumlupınar University, Evliya Çelebi Campus, 43100 Kütahya, Turkey*

^*^Corresponding author: fatihsen1980@gmail.com

^1^ These authours equally contributed this work.

**Materials**

Aldrich supplied superhydride, dimethylamine-borane, CoCl_2_, PdCl_2_ and activated carbon. C_2_H_5_OH and water used during this study were provided from Merck and Milli Q-pure machine, respectively. Before washing all glass pieces and other lab materials with large amount of distilled water, they were cleaned with acetone, then dried.

**Analytical investigations of Pd-Co@AC NPs**

TEM samples were made ready by dropping of 0.5 mg/mL ethanol solution of the prepared catalysts with an activated carbon support on a carbon covered 400-mesh copper grid, and then the solvent was vaporized. Elimination of excess solution was performed with an adsorbent paper and the sample was dried under vacuum at room temperature before analysis. TEM analysis was carried out by a JEOL 200 kV. More than 300 particles were calculated to get the integrated information about the overall distribution of Pd-based catalyst sample. During X-ray Photoelectron Spectroscopy (XPS) analysis, Specs spectrometer was employed and as an X-ray source Kα lines of Mg (1253.6 eV, 10 mA) was used. Sample preparation was done by depositing the catalyst on Cu double-sided tape (3M Inc.). C 1s line at 284.6 eV was selected as a reference point and all XPS peaks were fitted using a Gaussian function and the C 1s line at 284.6 eV was used as the reference line. XRD analysis were performed with a Panalytical Empyrean diffractometer with Ultima+theta–theta high resolution goniometer, having an X-ray generator (Cu K∞ radiation, k = 1.54056 Å ) and operating condition of 40 kV and 40 mA. UV–Vis analyses were taken by Perkin Elmer Lambda 750. 200–900 nm was selected to gather the data and 1 cm-long a quartz cell was employed. ^11^B NMR spectra were recorded on a Bruker Avance DPX 400 MHz spectrometer (128.2 MHz for ^11^B NMR).

**Investigation of performances of Pd-Co@AC NPs during DMAB dehydrogenation**

By means of the rate of hydrogen emission, the performance of Pd-Co@AC NPs during dehydrogenation of DMAB was examined. During the study, a coated vessel (50 mL) was put on stiring machine and kept the reaction temperature 25.0 ± 0.1◦C by flowing H_2_O in special parts of reaction vessel from a bath where temperature was fixed. After that, water was poured into a millimetric glass pipe (50 cm in height and 4.0 cm in size) and attached to the dehydrogenation chamber to find the amount of H_2_ generated. In a sample work, DMAB was transported into the reaction chamber with 25.0 ± 0.1◦C of fixed temperature. After transferring same amount of Pd-Co@AC NPs, dehydrogenation of DMAB was began by covering the reaction chamber and the amount of H_2_ fabricated was found writing H_2_O change in the column at 1000 round per minute. Also, by ^11^B NMR, the change of DMAB (δ= −12.6ppm) to (Me_2_NBH_2_)_2_ (δ = 5.0ppm) was discovered, besides measuring the amount of hydrogen gas.

**Reusability examination of Pd-Co nanomaterials stabilized by AC**

Some amount of AC-stabilized Pd-Co nanomaterials (0.3mM) was placed to get 20 mL of solution. This mixture and 100 mM DMAB was employed to measure the usage performance of AC-stabilized Pd-Co nanocatalyst during the reaction of DMAB at 25.0 ± 0.1◦C. Several experiments was carried out for this purpose. After the completion of DMAB change to metaborate, equal amount of DMAB was placed again to the reaction chamber suddenly. The data were written as % starting performance of Pd-Co nanomaterials stabilized by AC versus the number of catalytic cycles during DMAB dehydrogenation.





**Fig. S1.**UV-Vis absorption spectra of the aqueous solutions of Pd^+2^, Co^+2^, and Pd-Co@AC NPs.


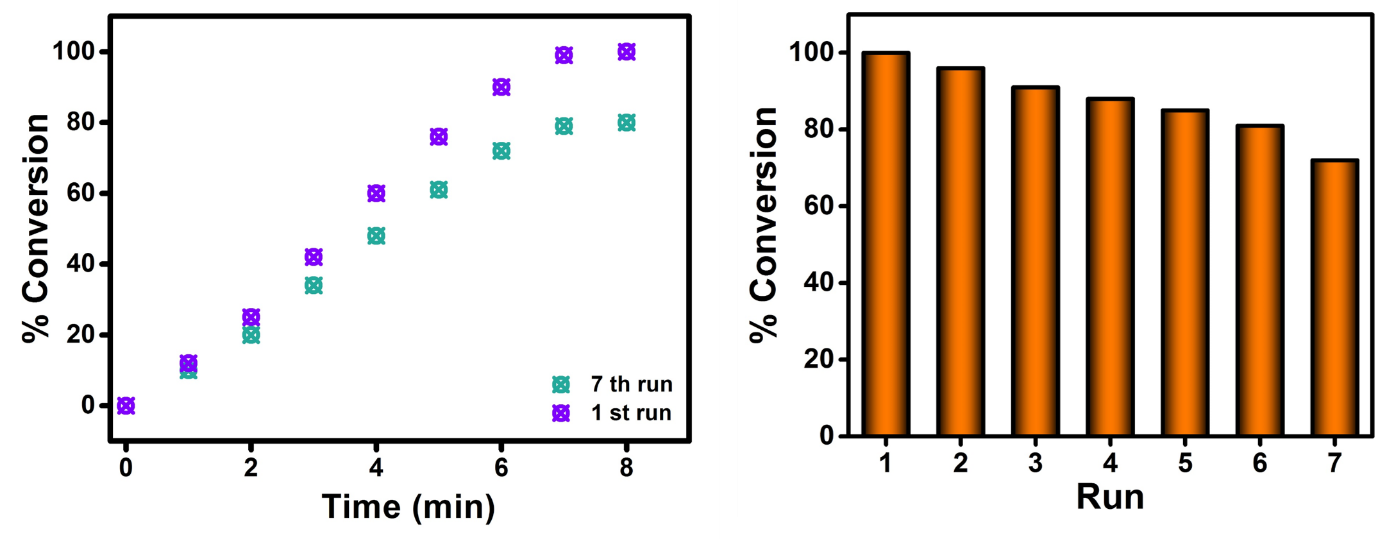


**Fig. S2.** Plots % conversion versus time graph for Pd-Co@AC NPs (7.5 % mol) catalysed dehydrocoupling of DMAB in THF at room temperature for 1^st^ and 7^th^ catalytic runs.

**% Conversion_average_ calculation:**

Catalyst

2(CH_3_)_2_NHBH_3_ [(CH_3_)_2_N.BH_2_)]_2_ + 2H_2_

RT

According to the reaction, 1 mol of H_2_ gas is released from 1 mol of DMAB. 2.250 x 10^-3^ mol DMAB was used. In this case, H_2_ gas is theoretically 2.250 x 10^-3^ moles.

Experimentally obtained H_2_ gas mol number:

P x V = n x R x T

P= Pressure (atm), (0.897 atm, Kutahya/Turkey)

V= Volume (L)

n= mol

R= Gas constant (0.08205 L.atm.mol^-1^. K^-1^)

T= Temperature (Kelvin) (25 °C= 298.15 K)

% Conversion calculation:

In 3 experiments, 65.0, 65.3, and 65.3 units of H_2_ gas released (1 unit= 0.94 mL).

Experiment 1

V_1_= 65.0 unit = 61.1 mL = 0.0611 L

P x V_1_ = n_1_ x R x T

(0.897 atm) x (0.0611 L) = n x (0.08205 L.atm.mol^-1^.K^-1^) x (298.15 K)

n_1_ = 2.240 x 10^-3^ mol (experimental data)

experimental data

X 100

% Conversion _1_ =

theoretical data data

X 100

2.240 x 10^-3^ mol

% Conversion _1_ =

2.250 x 10^-3^ mol

% Conversion _1_ = 99.6

Experiment 2 and 3

V_2,3_ = 65.3 unit = 61.38 mL = 0.06138 L

P x V_2,3_ = n_2,3_ x R x T

(0.897 atm).(0.06138 L) = n.(0.08205 L.atm.mol^-1^.K^-1^).(298.15 K)

n_2,3_ = 2.251 x 10^-3^ mol (experimental data)

X 100

experimental data

% Conversion _2,3_ =

theoretical data **data**

2.251 x 10^-3^ mol

X 100

% Conversion _2,3_ =

2.250 x 10^-3^ mol

% Conversion _2,3_ = 100.0

99.6 + 100.0 + 100.0

% Conversion _av_ =

3

% Conversion _av =_ 99.9

After 3 experiments, the average conversion rate was calculated as 99.9 ± 0.2.
